# LPS-Challenged Macrophages Release Microvesicles Coated With Histones

**DOI:** 10.3389/fimmu.2018.01463

**Published:** 2018-06-27

**Authors:** Rohini Ravindran Nair, Davide Mazza, Francesca Brambilla, Andrea Gorzanelli, Alessandra Agresti, Marco E. Bianchi

**Affiliations:** San Raffaele Scientific Institute, San Raffaele University, Milan, Italy

**Keywords:** histones, nucleosomes, macrophages, microvesicles, LPS, inflammation

## Abstract

Histones are the protein component of nucleosomes, which are the basic packing unit of chromatin. However, histones are also found in the blood, both as components of nucleosomes leaked out from dead cells, or expelled from neutrophils in the active process of NET formation. Circulating histones contribute to inflammation, and to lethality in sepsis, a hyperinflammatory condition, by interacting with specific receptors, notably toll-like receptor 4 (TLR4). Here, we show that histones are also actively released by LPS-activated macrophages in association with extracellular vesicles. Vesicle-associated histones can be recovered from the plasma of mice with sepsis. Actively released histones are on the outer surface of vesicles and can interact with TLR4. Thus, activated macrophages release histones without dying, at the same time, making their DNA more accessible and communicating to other cells that infection is present.

## Introduction

Histones package DNA by forming nucleosomes, the basic unit of chromatin. However, histones can be found outside cells, both as soluble proteins or as components of nucleosomes. In particular, blood often contains both nucleosomes and free histones, which can be used as biomarkers of various diseases. Nucleosomes are released by apoptotic cells, and the DNA they contain can provide sequence information about the cancer status of the cells that released it—the so called liquid biopsy. Histones are also released by neutrophils during the process of NET formation (1), whereby activated neutrophils eject their chromatin to trap pathogens and plug microvessels, preventing pathogen spreading (2).

Histones are found in abundance in the blood of patients and animal models undergoing sepsis, a hyperinflammatory condition, which is often fatal. Remarkably, histones act as inflammatory molecules, both by directly damaging endothelial cells (3), kidney tubular cells (4), and cardiomyocytes (5), and by acting as damage-associated molecular pattern (DAMPs) molecules that bind to pattern recognition receptors and induce target cells to release further inflammatory mediators. In particular, free histones activate toll-like receptor 4 (6) and promote the release of chemokines and cytokines by inflammatory cells (4), contributing to hyperinflammation in sepsis. Indeed, injection of free histones produces a sepsis-like condition in mice and baboons, and neutralization of histones with anti-histone antibodies or injection of activated protein C (APC), a serine protease that cleaves histones, prevents lethality in sepsis (3).

Whereas neutrophils and dying cells certainly release chromatin, nucleosomes, and histones, other unknown processes may mediate the release of histones from cells. We previously showed that activation of macrophages with LPS induces a rapid loss of histones from the cell nuclei (7). Here, we set out to investigate the fate of the histones lost from activated macrophages and found that they are released in the extracellular medium. Remarkably, at least part of the released histones is associated to extracellular vesicles (EVs). EVs are small membrane vesicles that comprise apoptotic bodies [also called macrobodies (MBs), 0.5–2 µm in diameter] that bleb from apoptotic cells, microvesicles (MVs) (50–1,000 nm) that bud off from the plasma membrane, or exosomes (40–120 nm) that derive from multivesicular bodies (8). Most, if not all, cell types release EVs, which carry a subset of proteins, lipids, and nucleic acids that are derived from the parent cell. EVs can transfer their contents to target cells in a specialized form of intercellular communication, including to, from and between immune cells (9). We found that extracellular histones from macrophages are exposed on the outer surface of EVs and can interact with receptors (notably, TLR4) of target cells, promoting inflammatory responses. Histone-bearing EVs released from macrophages can be retrieved from the plasma of mice challenged with lethal doses of LPS, suggesting that they can contribute to sepsis.

## Materials and Methods

### Animals

Animals were anesthetized by intraperitoneal (i.p.) injection of Avertin (T48402, 2,2,2-Tribromoethanol 97%, Sigma-Aldrich) and euthanized by CO_2_ inhalation. Male and female mice were used and always gender-matched in each experiment.

*C57/Bl6*: 6- to 8-week-old mice were purchased from Charles River.

*Tlr4^−/−^ mice (C57BL/6)* were purchased from Jackson Laboratories and bred in-house.

*H2B-GFP mice* obtained by Jackson laboratory were produced by Hanno Hock’s lab (10). These R26-M2rtTA; Col1a1-tetO-H2B-GFP compound mutant mice are perfectly viable and allow doxycycline-inducible synthesis of histone H2B-GFP in all tissues, including macrophages from the bone marrow.

### Materials

Exosome-free FBS was prepared by centrifuging aliquots of 30% FBS in DMEM (GIBCO) at 100,000 *g* at 4°C for 16 h.

### Primary Cells

Bone marrow-derived macrophages (BMDM) were isolated as previously described (11). Six- to 8-week-old C57/Bl6 mice (Charles River Laboratories, Calco, Italy) were sacrificed and both femurs were separated from adherent tissue. The ends of the bones were cut off and the marrow tissue was flushed by irrigation with PBS. The marrow plugs were dispersed by passing through a 25-G needle, and the cells were suspended by pipetting and recovered by centrifugation. The cells obtained from five mice were pooled and resuspended in 40 ml DMEM containing 10% exosome-free FBS, 1% penicillin/streptomycin, 1% glutamine, and 50 ng/ml recombinant murine granulocyte-macrophage colony-stimulating factor (GM-CSF) (PeproTech, Rocky Hill, NJ, USA) and distributed in four 100 mm dishes for bacterial cultures (Sterilin Ltd., Cambridge, UK) to minimize cell adhesion. At day 3 of culture, the medium in the plates was doubled and GM-CSF added; at day 6, BMDMs were re-plated in cell culture dishes (1–2 × 10^5^ cells/cm^2^) for terminal differentiation. The cells were incubated at 37°C in a humidified atmosphere containing 5% CO_2_ and 5% oxygen.

Bone marrow-derived macrophages were isolated also from H2B-GFP mice that were fed with water containing 2 mg/ml of doxycycline and 5% sucrose for 3 weeks before sacrificing them for bone marrow collection. BMDMs were cultured as described above in the absence of doxycycline to exclude the presence of newly synthesized H2B-GFP in the cytoplasm at the time of vesicle collection.

To perform all the experiments cited in the paper, cells were either left untreated or challenged for 4 h with 100 ng/ml Salmonella typhimurium LPS (Sigma-Aldrich, St. Louis, MO, USA). Where indicated, 10 µM MG132 was added to cells 1 h before LPS.

### Quantitative Western Blotting

Bone marrow-derived macrophagess were lysed in SDS-PAGE loading buffer without Bromophenol Blue, and the Quant-iT PicoGreen dsDNA kit (Invitrogen, Carlsbad, CA, USA) was used to measure their DNA content, as described (7, 12). For histone quantification, the lysate equivalent to 150 ng DNA was loaded into each well of 15% SDS-PAGE gels. Gels were run at constant 100 V and transferred to nitrocellulose membranes with the Trans-BlotTurbo^™^ Blotting System (Bio-Rad, Hercules, CA, USA). Filters were blocked with 2% ECL Prime blocking agent (GE Healthcare Life Sciences, Schenectady, New York, NY, USA) in Tris-buffered saline pH 7.0, 0.1% Tween 20 (TBS-T). Blocked membranes were probed with the following antibodies in TBS-T at 4°C for 16–24 h: rabbit Chip-Grade anti-H3 antibody (1:1,000; ab1791, Abcam plc, Cambridge, UK), anti-H2A (1:1,000; ab18255, Abcam plc, Cambridge, UK), anti-H2B (1:250; Upstate #07-371, Merck Millipore, KGaA, Darmstadt, Germany), mouse anti-beta-actin (1:2,000; A5441, Sigma-Aldrich, St. Louis, MO, USA), anti-flotillin (1:250; #610820, BD Bioscience, San Jose, CA, USA), anti-TSG101 (1:1,000; ab30871, Abcam plc, Cambridge, UK), anti-citrullinated H3 (1:1,000; ab5103, Abcam plc, Cambridge, UK), anti-HLA IA/IE-PE (BD PharmingenTM).

Membranes were washed and detected with anti-rabbit ECL Plex Goat-anti-rabbit IgG-Cy5 (PA45011, 1:2,000, GE Healthcare, Little Chalfont, UK) at room temperature for 1 h. Sixteen-bit images were acquired with a Typhoon FL9000 (GE Healthcare; 633 nm); signals were within the linear part of the dynamic range. Quantification of Western blot signals was performed with IMAGEJ software (13).

For the analysis of histone modifications, EVs and soluble fractions were resuspended in RIPA buffer containing protease inhibitor cocktail (Sigma-Aldrich, St. Louis, MO, USA). Protein quantification in samples was performed using the BCA quantitation kit (Thermofisher, Waltham, MA, USA). Samples containing 10 µg of total vesicle proteins or 100 µg of soluble proteins were loaded onto SDS-PAGE gels and analyzed by western blotting as described above with the following antibodies diluted 1:1,000: anti-H3K4me3 (ab8580), anti-H3K36me3 (ab9050), anti-H3K9me3 (ab8898) (Abcam plc, Cambridge, UK).

### Stochastic Optical Reconstruction Microscopy (STORM) Imaging

#### Immunostaining

Cells were plated on 8-well Lab-tek chambered no. 1 coverglass (Nunc, Waltham, MA, USA) at a seeding density of 1–2 × 10^5^ cells/cm^2^, treated or not with LPS for 4 h, fixed with 4% PFA in PBS for 10 min, and then permeabilized with 0.3% v/v Triton X-100 (Sigma) in PBS for 10 min at room temperature. After 1 h incubation at room temperature with blocking buffer containing 10% (wt/vol) BSA (Sigma) in PBS, cells were incubated overnight with the primary antibody diluted 1:50 in blocking buffer containing 10% normal goat serum, 0.05% Tween 20 (Sigma), and 2% BSA, and then for 40 min with the appropriate dilution of dye-labeled secondary antibodies. Repeated washing were done at every step with 0.1 M glycine (Pan Reac Applichem, Gatersleben, Germany) containing 0.2% BSA.

#### Primary Antibodies

Rabbit polyclonal anti-H2A (Abcam ab18255) and donkey-anti rabbit (711-005-152; Jackson ImmunoResearch) secondary antibodies were labeled in-house with different combinations of pairs of activator/reporter dyes (14). Briefly, dyes were purchased as NHS ester derivatives: Alexa Fluor 405 Carboxylic Acid Succinimidyl Ester and Alexa Fluor 647 Carboxylic Acid Succinimidyl Ester (Invitrogen). Labeling reactions were performed by incubating at room temperature for 40 min a mixture containing the secondary antibody in 0.12 M NaHCO_3_, and the appropriate pair of activator/reporter dyes diluted in DMSO. Purification of labeled antibodies was performed using NAP5 Columns (GE HealthCare). The dye to antibody ratio was quantified using Nanodrop and only antibodies with a composition of 3–4 Alexa Fluor 405 and 0.9-1.2 Alexa Fluor 647 per antibody molecule were used for imaging.

Extracellular vesicles in H2B-GFP BMDMs were analyzed by STORM with the same protocol without the permeabilization step to avoid excessive staining of the nucleus. The cell membranes were stained with 50 µg/ml Concanavalin A-TRITC (Molecular Probes, Eugene, OR, USA).

#### Imaging

Stochastic optical reconstruction microscopy imaging was performed on a Leica SR GSD (Leica Microsystems SRL, Milan, Italy) super-resolution microscope equipped with a 160×, 1.43 NA objective, Andor iXon Ultra-897 EM-CCD camera and three (405 nm, 30 mW; 488 nm, 300 mW, and 642 nm, 500 mW) solid-state lasers. The Lab-tek chamber was mounted on the microscope stage and the medium was substituted before acquisition to a mix of Glucose oxidase (560 µg/ml), catalase (400 µg/ml), and Cysteamine HCl (10 mM) in PBS with 10% glucose w/v at pH 8 in order to induce fluorophore blinking (15). The medium was replaced with fresh one after each acquisition. The acquisition was carried out as follows. First, a reference image was collected using the 642 nm laser at low power (AOTF set to 5%). Next, the 642 nm laser power was tuned up to 80% and the sample was illuminated for 500 frames (8 ms exposure) in order to push most of the dye molecules in the long-lived dark states. Finally, the laser power was tuned back down to 60% and 40,000 frames were collected to image the individual dyes that spontaneously revert back to the fluorescent state. The individual molecules were localized using the proprietary Leica analysis software and discarding all the detected events with less than 20 photons/pixel. When necessary, the lists of single-molecule localizations were corrected for drift. To avoid over-counting of molecules appearing for multiple frames, localizations in consecutive frames within 50 nm were merged together. The localization list was then exported in an ASCII file for further analysis.

#### Cluster Quantification From STORM Images

Super-resolution images were rendered with a 20 nm pixel size using the Leica proprietary software. Analysis of the histone clutches from the super-resolution images was performed as previously described (16) using the FindCluster algorithm developed in Matlab by Dr. Lakadamyali. Briefly, the software uses the localization lists to construct images with a 10 nm pixel, which are then convolved with a 5 × 5 square kernel to generate a density map. A threshold is then used to binarize the density image (set to 0.006 nm^2^), in order to identify the clusters and discard isolated localizations that do not belong to histone clusters. Next, segmentation by connected component analysis of the binary image is performed, and on each individual connected component iterative identification of sub-clusters allows the measurement of the position of the clutches, of the number of single-molecule localizations belonging to each clutch and for the size of each clutch, defined as the spread of the localizations (SD) belonging to the same clutch. Non-parametric Kolmorogov–Smirnov tests were used for comparing clutches properties upon different experimental conditions.

### Isolation of EVs

For each isolation, BMDMs derived from five mice were pooled and 5 × 10^7^ cells were plated in exosome-depleted medium. Cell-conditioned media (CCM) were harvested and exosome purification was performed as previously described (17). CCM were centrifuged at 300 *g* for 5 min to eliminate floating cells, followed by centrifugation at 2,000 *g* for 20 min to pellet MBs. Then, 70 ml CCM were concentrated to 500 µl with Centricon Plus-70 Centrifugal Filters (Millipore, Billerica, MA, USA, Ultracel-PL Membrane, 10 kDa, UFC701008) using an Eppendorf centrifuge (Hamburg, Germany) at 3,500 *g* at 4°C. The concentrated CCM was then recovered with a reverse spin at 1,000 *g* for 2 min. Simultaneously, a discontinuous iodixanol gradient was prepared by diluting a stock solution of 60% w/v OptiPrep™ (Axis Shield Diagnostics, Dundee, UK) with 0.25 M sucrose, 10 mM Tris, pH 7.5. A 40, 20, 10% (3 ml each) and 5% (2.5 ml) iodixanol gradient was formed in 14 × 89 mm Ultra-Clear™ Beckman Coulter centrifuge tubes. Concentrated CCM (500 µl) was overlaid on the iodixanol gradient and centrifuged using a SW 41 Ti rotor for 16 h at 100,000 *g* (*k*-factor: 277.5) at 4°C. Fractions of 1 ml were collected from the top of the gradient, and 50 µl from each fraction were analyzed by western blotting. For analytical preparations, fractions 1–5, containing soluble proteins, and fractions 6–9, containing EVs, were each diluted to 10 ml in PBS and centrifuged at 100,000 *g* for 3 h at 4°C in a SW41Ti rotor. The resulting pellets were either resuspended in 100 µl RIPA buffer for protein quantitation or in PBS for flow cytometry and electron microscopy.

For functional studies, pellets were resuspended in PBS and their protein content quantified as described above.

#### Electron Microscopy of EVs

Extracellular vesicles were visualized using transmission electron microscopy (17). For EV negative staining, 2 µl of the EV-containing fraction were transferred onto each of two Formvar-carbon coated electron microscopy grids (Electron Microscopy Sciences, Hatfield, PA, USA) that were previously glow-discharged for 30 s. The grids were kept wet on the side of the membrane during all steps, but dry on the opposite side. After 5 min, grids were blotted on filter paper, washed in PBS, and transferred onto a 50 µl drop of 1% glutaraldehyde for 5 min and then onto a 100 µl drop of distilled water for 2 min. This was repeated five times for a total of six water washes. To contrast the samples, grids were transferred to a 50 µl drop of 2% uranyl-oxalate solution, pH 7, for 2 min, then blotted gently on Whatman no.1 filter paper, and air dried.

For EVs immunolabeling, EVs adsorbed on Formvar-carbon coated grids were fixed in a 50 µl drop of 4% paraformaldehyde (Electron Microscopy Sciences) for 20 min. After several washes in PBS, free aldehyde groups were quenched in 0.15% glycine in PBS (2 min washes repeated three times) and transferred to blocking buffer (1% BSA in PBS) for 5 min. Samples were then incubated with primary antibody diluted in blocking buffer for 30 min, washed five times 2 min each in 0.1% BSA in PBS, and incubated with 5 nm protein A gold (Utrecht University, Netherlands). After several washes in PBS, grids were transferred to a 50 µl drop of 1% glutaraldehyde for 5 min before transferring to a 100 µl drop of distilled water for 2 min. This was repeated five times for a total of six water washes. To contrast the samples, grids were transferred to a 50 µl drop of 2% uranyl-oxalate solution, pH 7, for 5 min before transferring to a 50 µl drop of methylcellulose-UA (a mixture of 100 µl of 4% uranyl acetate and 900 µl of 2% methylcellulose) for 10 min, placing the grids on a glass dish covered with Parafilm on ice. The grids were removed with stainless steel loops and excess fluid blotted gently on Whatman no.1 filter paper. Grids were left to dry and stored in appropriate grid storage boxes.

Grids were observed with a Zeiss LEO 512 transmission electron microscope. Images were acquired by a 2k × 2k bottom-mounted slow-scan Proscan camera controlled by EsivisionPro 3.2 software.

#### Measurement of Total and EV-Associated Histones in Blood

We injected 8- to 12-week-old BALB/c mice (Charles River Laboratories, Calco, Italy) with 2 and 10 mg/kg LPS (*n* = 9) or control PBS (*n* = 8) in the tail vein (3). At the indicated times (1 and 20 h postinjection), we collected blood from the retroorbital sinus of the sedated mice and obtained plasma, which was either analyzed by Western blotting, or for the presence of EVs with beads coated with anti-CD63. We verified that the beads were not saturated and that the number of positive beads was proportional to the amount of EVs by incubating a constant amount of beads with decreasing amount of plasma (Figure S3 in Supplementary Material).

#### Trypsin Treatment of EVs

Extracellular vesicles (10–20 µl) were suspended in 100 µl of 2.5 mg/ml trypsin in PBS (Lonza, Basel, Switzerland), or trypsin plus 0.5% Triton X-100 (Sigma), and incubated at 37°C for 60 min. Samples were then analyzed by Western blotting.

#### AnnexinV and Propidium Iodide (PI) Staining

5 × 10^6^ BMDMs were plated in 60 mm bacterial plates and left untreated or challenged with 50 ng/ml LPS for 4 h. BMDMs treated with cyclohexamide (1 µg/ml) and TNF-α (2 ng/ml) were used as positive control for apoptosis. The medium containing cells growing in suspension was transfer to a tube while adherent cells were detached by gentle pipetting in Versene (0.5 mM EDTA in PBS) and added to the same tube. Cells were then washed once with Annexin V binding buffer (10 mM Hepes pH 7.4, 140 mM NaCl, and 2.5 mM CaCl_2_) and resuspended in 100 µl of the same buffer. AnnexinV—APC conjugated (5 µl; Biolegend) was added to the cells and after 15 min of incubation in dark PI (Sigma; 1 µg/ml) was added. Cytofluorimetry was performed with the Accuri™ FACS (Becton Dickinson).

#### Apoptosis Quantification by TUNEL Assay and Imaging

Tunel assay was performed using the “DeadEnd™ Fluorimetric TUNEL System” (Promega) and following manufacturer’s instructions. Briefly, cells were grown on coverslips and fixed with 4% formaldehyde after the treatments described before. After Triton X-100 permeabilization, cells were pre-equilibrated in equilibration buffer. DNA strand breaks were labeled by TdT enzyme with Fluorescein-12dUTP for 1 h at 37°C in the dark. Nuclei were counterstained with Hoechst 33342 and coverslips mounted on glass slides. Images were acquired by confocal microscopy as described below.

#### Cytotoxicity Assay

The CytoTox 96 Non-Radioactive Cytotoxicity Assay, Promega was used following manufacturer’s instruction. Briefly, BMDMs were plated at 10^5^ cells/well in triplicate in 96-well culture plate. Recommended controls were included in each experiment. After LPS treatment, 50 µl aliquots from each well were transferred to a fresh 96-well flat clear bottom plate and 50 µl of the CytoTox 96 reagent were added to each well. The plate was covered with adhesive foil and incubated for 30 min at room temperature in the dark. 50 µl of stop solution blocked the reaction and the absorbance was recorded at 490 nm using a plate reader (iMark Microplate Absorbance Reader, Biorad, 1681135). Background-subtracted absorbance values were then used to calculate relative cytotoxicity using the following formula:

Relative Cytotoxicity=(Experimental LDH Release, OD490)/(Maximum LDH Release, OD490) × 100.

#### Flow Cytometry of EVs

Samples (in triplicate) in 1.5 ml tubes according to the manufacturer’s instructions (Thermofisher Scientific, Waltham, MA, USA) were prepared by diluting the purified EVs in 495 µl of 0.22 µm filtered PBS and adding 5 µl of 0.5 mM CFSE (or DMSO as diluent control), and incubated for 30–45 min at 37°C in the dark. Esterases present both in cell cytoplasm and in secreted EVs cleave the acetate groups from CFSE that now have a peak excitation at 494 nm and a peak emission at 521 nm, which we measured using 488 nm (blue) laser excitation and 535/35 band pass filter for detection.

Extracellular vesicles (30 µg of protein) from H2B-GFP BMDMs, either unstimulated or LPS challenged, were incubated for 16 h with anti-CD63-coated latex beads (4 µm, Molecular Probes). Beads were washed with 2% BSA in PBS and analyzed for H2B-GFP fluorescence using a Beckman Coulter’s Gallios flow cytometer.

#### Stimulation of Naïve Macrophages With Free Histone, Apoptotic Bodies, and Microvesicles and Exosomes (MEs)

Microvesicles and exosomes were isolated as described before from untreated and LPS-stimulated cells. MEs (100 µl per well) were added to wells of 6-well plates; each well contained 2 × 10^6^ BMDMs in 1 ml DMEM medium supplemented with Polymyxin B (PMB) (10 µg/ml). Treatment was done for 4 h at 37°C in a 5% CO_2_ atmosphere, after which cells were lysed. This was followed by RNA isolation, cDNA preparation, and RT-PCR for inflammatory genes.

Where indicated, we incubated 100 µl MEs with 10 µg anti-pan-histone antibody (anti-histone clone H11-4, Millipore) for 2 h at 4°C, before adding them to wells where BMDMs had been incubated with 10 µg of “Mouse BD FC Block,” a purified rat anti-mouse CD16/CD132 mAb (BD Bioscences), to block Fc-gamma receptors.

RNA was extracted from BMDMs with the Illustra RNAspin Mini RNA Isolation Kit (GE healthcare). DNA was removed through DNase treatment as described by the manufacturer. One microgram of total RNA was reverse-transcribed using random exanucleotides and SuperScript II reverse transcriptase (18418-012 and 18064-071, respectively; Life Technologies, CA, USA) as described by the manufacturer. cDNAs were amplified by real-time PCR on an iQ5 instrument (Roche, Basel, Switzerland), using the relative quantification software, with the iTaqTM Universal SYBR^®^ Green Supermix (Bio-Rad). The following primers were used:

Tnf (Forward 5′-cttctcattcctgcttgtgg-3′, Reverse 5′-cgagagaggaggttgactttc-3′);
Il1b (Forward 5′-gctacctgtgtctttcccgtg-3′, Reverse 5′-gggtgtgccgtctttcattac-3′);
Il6 (Forward 5′-ctgggaaatcgtggaaatgag-3′, Reverse 5′-ctctggctttgtctttcttgttatc-3′);
Ikbia (Forward 5′-cttggctgtgatcaccaaccag-3′, Reverse 5′-cgaaaccaggtcaggattctgc-3′)

Results are expressed as fold change of expression in treated samples compared to untreated samples after normalization for ribosomal protein S29 (Rps29) gene expression (Forward 5′-agcatgttcggttccacttg-3′, Reverse 5′-agtcacccacggaagttcg-3′).

## Results

### Macrophages Challenged With LPS Lose Histones From the Nucleus and Modify Their Chromatin Architecture

**Figure 1:**
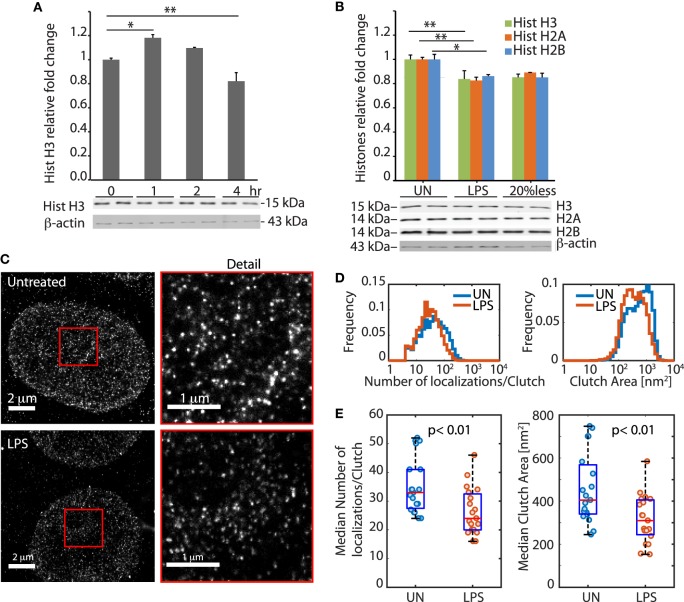
LPS-challenged bone marrow-derived macrophages (BMDMs) lose histones from the nucleus and modify their chromatin architecture. **(A)** Histone H3 quantification in BMDMs at 0, 1, 2, and 4 h after LPS challenge. **(B)** Histone H3, H2A, and H2B quantification in untreated and LPS-treated BMDMs. The bar plots in **(A,B)** show the fluorimetric quantification of the bands that were normalized to beta-actin, and by setting to “1” the histone amount in BMDMs not challenged with LPS. Error bars represent SDs. One-way ANOVA with Dunnett’s posttest; *p < 0.05; **p < 0.01. In each panel, one representative experiment in duplicate, of three performed, is shown. **(C)** Representative stochastic optical reconstruction microscopy images of H2A in untreated and LPS-challenged BMDMs. The red squares are enlarged to show the detail. **(D)** Distributions of the number of H2A localizations per clutch, and of clutch area, in untreated and LPS-challenged BMDMs (*n*_cells_ = 11 per group; *n*_clutches_ = 18,761 and 11,405 for untreated and LPS-challenged cells, respectively). Both distributions show significant differences between untreated and LPS-challenged BMDMs (*p* < 10^−15^, two-sample Kolmogorov–Smirnov test). **(E)** Difference between the medians of the distributions in single cells (*n* = 11 for exp 1, and *n* = 8 for exp 2). Each dot represents one cell. The groups of untreated and LPS-challenged BMDMs are statistically different, both for clutch area and number of localizations per clutch (*p* < 0.01, two-sample *t*-test, after testing for normality by one-sample Kolmogorov–Smirnov test).

We exposed BMDMs to 100 ng/ml LPS and measured the cellular content of histone H3 over time. Histone H3 increased slightly during the first hour and then decreased markedly over time, reaching a minimum after 4 h that corresponded to a loss of about 20% of histone H3 relative to unstimulated cells (Figure 1A), as previously described (7). The levels of histones H2A and H2B were also reduced by about 20% after exposure to LPS (Figure 1B).

More than 99% of total cell histones are associated to DNA to form chromatin, and a decrease of 20% in histone content is expected to alter the global chromatin conformation inside the nucleus of the cell. To assess global chromatin conformation, we imaged BMDM nuclei by STORM super resolution microscopy. STORM images revealed striking changes in the distribution of histone H2A inside the nuclei of BMDMs exposed to LPS for 4 h. Histone H2A appears clustered in discrete and spatially separated nanodomains called clutches (16), which represent assemblages of several nucleosomes. We observed that the distributions of the number of localizations per clutch (which is proportional to the number of H2A molecules per clutch) and clutch area were significantly different in BMDMs challenged or not with LPS (Figures 1C–E).

Taken together, these experiments show that LPS-challenged BMDMs lose histones from their nuclei, and rearrange their chromatin accordingly.

### Histones Lost From LPS-Challenged Macrophages Are not Degraded but Released into the Extracellular Medium

**Figure 2:**
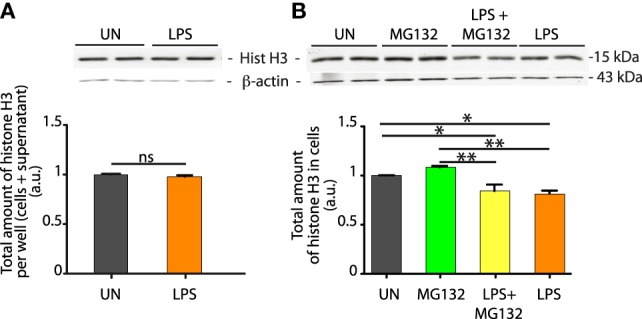
Histones lost from LPS-challenged bone marrow-derived macrophages (BMDMs) are not degraded. **(A)** The total content of the culture well, comprising both BMDMs and their supernatant, was lysed in hot SDS-PAGE loading buffer, and histone H3 was quantitated by western blotting. Significance was assessed with a *t*-test. This experiment was repeated four times. **(B)** Histone H3 quantitation in BMDMs after LPS challenge, in the presence or absence of the proteasome inhibitor MG132. H3 quantitation and representation of the results as in Figure 1. One-way ANOVA with Tukey’s posttest; *p < 0.05; **p < 0.01.

Histones removed from the cell can follow two possible routes: degradation or extracellular release. If histones are simply shuffled from the nucleus to the extracellular medium, the total histone content of the cells plus their medium should hold constant. We then dissolved in hot SDS-PAGE denaturation buffer the total content of each well, comprising the cells plus the medium bathing them, and quantitated histone H3 by Western blotting. We found no significant difference in total H3 histone content (Figure 2A).

Histone degradation is proteasome-dependent (18) and, therefore, we incubated LPS-exposed BMDMs with the proteasomal inhibitor MG132. The content of histone H3 was not different in cells treated or not with MG132, and still did significantly decrease in LPS-exposed BMDMs in the presence of MG132 (Figure 2B). As a positive control for the efficacy of MG132, we assessed the transcription profile of inflammatory genes using real-time PCR. We observed that MG132 did prevent the transcriptional activation of NF-κB dependent genes (Figure S1 in Supplementary Material), as expected from the inhibition of the proteasomal degradation of IκBα.

Taken together, these experiments indicate that a significant fraction of histones of LPS-exposed BMDMs are released into the medium bathing the cells.

### Histones Are Released As Both Soluble and Vesicle-Associated Molecules

**Figure 3:**
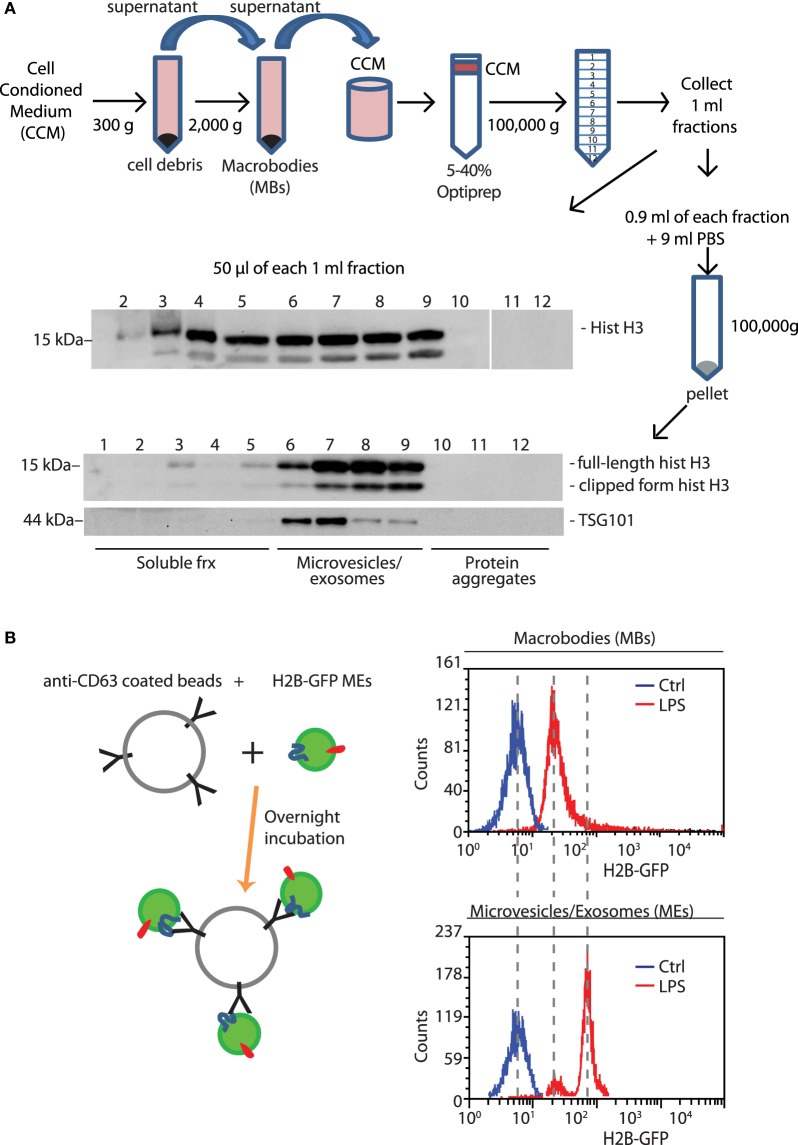
Histones lost from LPS-challenged BMDMs are released into the medium, in soluble and EV-associated forms. **(A)** The supernatant of BMDMs challenged with LPS for 4 h was collected, centrifuged to eliminate cell debris, and then large vesicles that were retrieved separately [macrobodies (MBs)]. The concentrated cell-conditioned medium (CCM) was then loaded onto a discontinuous Optiprep density gradient. Twelve fractions were collected, and an aliquot was tested by Western blotting for the presence of histone H3; the remainder was centrifuged, and the pellets were dissolved in hot SDS-PAGE loading buffer. Microvesicles and exosomes (MEs)-containing fractions were identified by the presence of TSG101, a membrane marker (19). One representative experiment, of four performed, is shown. **(B)** Extracellular vesicles in supernatants of H2B-GFP-expressing BMDMs were trapped by beads coated with anti-CD63 antibodies; beads were analyzed for GFP fluorescence by flow cytometry. MEs contain distinctly more H2B-GFP than macrobodies (*n* = 3, *t*-test, *p < 0.05).

Proteins released from cells can be either soluble or associated to EVs. We used differential centrifugation followed by Optiprep density gradient centrifugation (17) to assess whether histones released by BMDMs are soluble or vesicle-associated (Figure 3A).

Supernatants of LPS-activated BMDMs were collected, centrifuged at low speed to remove cell debris, and re-centrifuged at 2,000 *g*. The pellet was resuspended and analyzed by flow cytometry and electron microscopy (Figure S2 in Supplementary Material). This fraction contained MBs of 0.5–1 µm in diameter and aggregates of small vesicles.

The supernatant of samples centrifuged at 2,000 *g* was concentrated and subjected to density gradient centrifugation on a 5–40% Optiprep gradient. Twelve fractions of 1 ml were collected and an aliquot was analyzed by Western blotting (Figure 3A, middle panel). Fractions from 3 to 9 scored positive for histone H3. Subsequently, fractions were diluted and centrifuged again to recover both microvesicles and exosomes (MEs), which we collectively call MEs (Figure 3A, middle panel). Fractions 6–9 contained vesicles of 20–200 nm in diameter (Figure S2 in Supplementary Material) and were positive for both histone H3 and TSG101, a marker of EVs. Fractions 1–3 correspond to soluble proteins and did not contain TSG101.

In vesicular fractions, two bands were detected by anti-histone H3 antibodies; the lower band corresponds to histone H3 whose N-terminal tail has been proteolytically cleaved (20).

To confirm the existence of histones associated to EVs, we used BMDMs derived from H2B-GFP knock-in mice (10). These BMDMs, whose nuclei contain H2B-GFP, were exposed or not to LPS. Their conditioned media were centrifuged first at low speed, to recover MBs, and then at high speed to recover MEs. Resuspended MBs and MEs were incubated with beads armed with antibodies against CD63 (Lamp-3), a marker of endolysosomal membranes (21). Flow cytometry documented the presence of fluorescent beads, indicating that H2B-GFP was associated with vesicular membranes (Figure 3B).

Taken together, these experiments show that LPS-challenged BMDMs translocate histones to the extracellular medium, both as soluble proteins, or associated to vesicles.

### Vesicle-Associated Histones Do Not Originate From Dead Cells

Histones are released passively from necrotic and apoptotic cells, and we thus wondered whether vesicle-associated histones derived from dead cells. Super-resolution analysis of BMDM nuclei after LPS exposure was not indicative of cell death: apoptotic nuclei would appear condensed, and necrotic nuclei would appear swollen and diffuse; we only scored a few percent of nuclei with these morphologies (data not shown).

**Figure 4:**
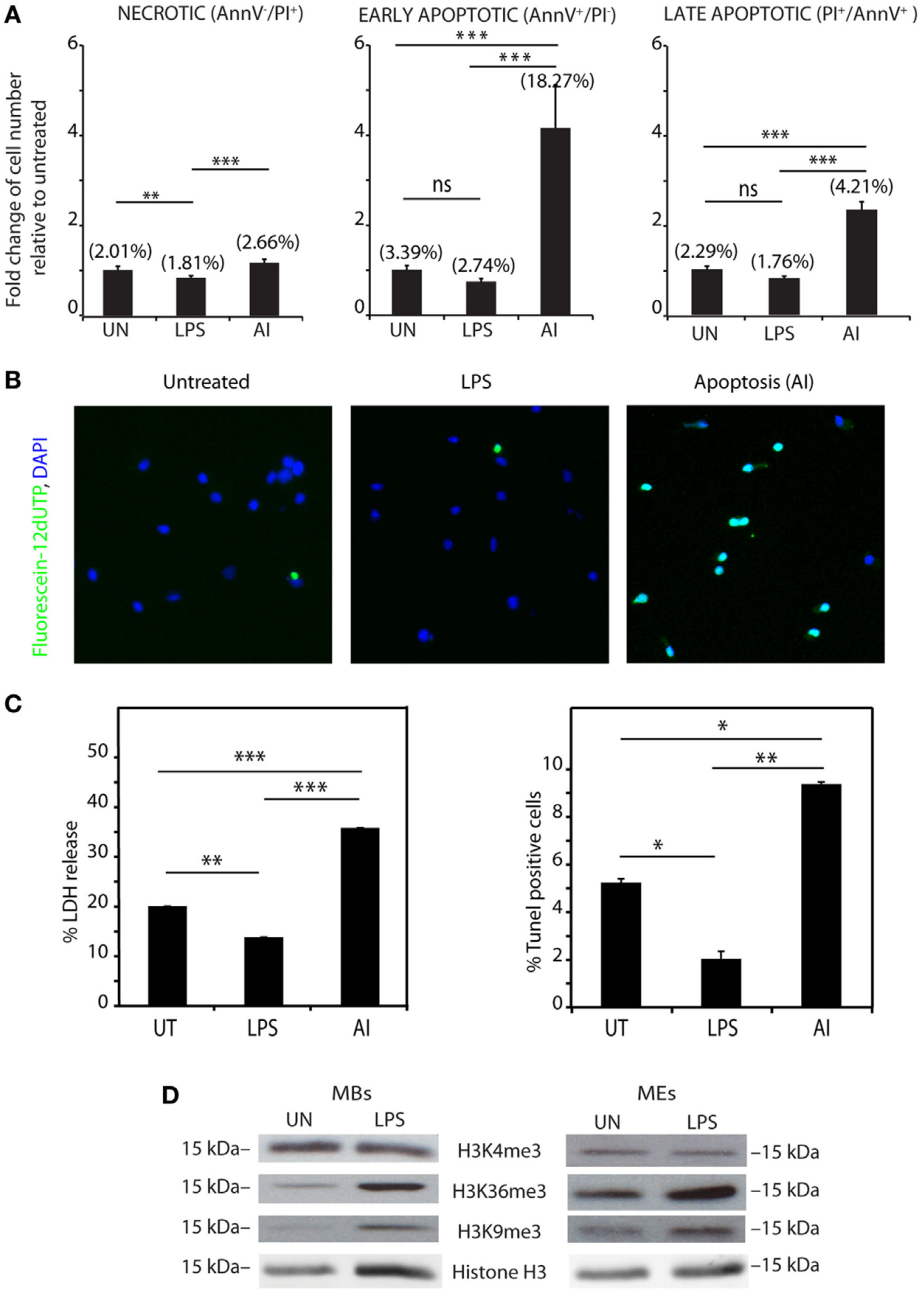
Histones are actively rather than passively released. **(A)** Bone marrow-derived macrophages (BMDMs), either untreated, LPS-challenged, or induced to apoptose (AI) with TNF-α and cycloheximide (positive control), were stained with annexin V-AlexaFluor and propidium iodide (PI) and analyzed by flow cytometry. The numbers between parentheses show the actual fraction of cells in the population. The bars show the number of dead cells, relative to the untreated samples. Double negative cells are alive (not shown), annexin V^–^ PI^+^ cells are necrotic (left panel), annexin V^+^ PI^–^ cells are early apoptotic (middle panel), and double positive cells are late apoptotic (right panel). **(B)** TUNEL staining of BMDMs, challenged or not with LPS, or induced to apoptose, and counterstained with DAPI. Fluorescein-12-dUTP incorporation results in localized green fluorescence in the nucleus of apoptotic cells only. **(C)** LDH release and percentage of TUNEL-positive BMDMs challenged or not with LPS, or induced to apoptose. Complete (100%) LDH release was obtained by lysing BMDMs with 1% Triton X-100. Data are expressed as a percentage of complete LDH release, after subtraction of background (serum medium not incubated with cells). In panels **(A–C)**, data represent one of four different experiments with similar results (each with *n* = 3 per group, one-way ANOVA plus Tukey’s posttest, *p < 0.05, **p < 0.01, ***p < 0.001). Error bars represent SDs. **(D)** Histone marks H3K4me3, H3K36me3, and H3K9me3 in macrobodies and microvesicles and exosomes isolated from untreated and LPS-challenged BMDMs.

However, to directly test a dead-cell origin of vesicle-associated histones, we determined the percentage of dead BMDMs, either LPS-challenged or not; as a positive control, we exposed BMDMs to 2 ng/ml TNF-α and 1 µg/ml cycloheximide. The fraction of dead cells was evaluated by flow cytometry using annexin V and PI staining. The percentage of annexinV-positive, PI-positive, and annexinV-PI double positive BMDMs was lower in BMDMs exposed to LPS (Figure 4A) than in unexposed cells. We also performed TUNEL assays (Figure 4B) and measured cytotoxicity as released LDH (Figure 4C). Both assays confirmed that LPS stimulation promotes survival of BMDMs, as described previously (22).

Since exposure to LPS decreases the number of dead BMDMs, we infer that extracellular histones are not released passively by dead BMDMs.

### Vesicle-Associated Histones Originate From the Nucleus

We considered the possibility that extracellular histones do not originate from the nucleus, but leak out from the cytoplasm soon after ribosomal synthesis. To test this possibility, we analyzed the posttranslational modifications of histone H3 associated to vesicles. Trimethylation of histone H3 to H3K4me3 and H3K36me3 occur only on the promoter and gene bodies of transcribed genes, whereas H3K9 trimethylation occurs only in heterochromatin; all these modifications occur only in the nucleus. We found that both the MB and ME fractions contain H3 modified at K9 and K36 (Figure 4D), which is compatible with a nuclear origin of least a part of extracellular histones.

### Mice Challenged With LPS Have Histone-Laden Vesicles in Plasma

**Figure 5:**
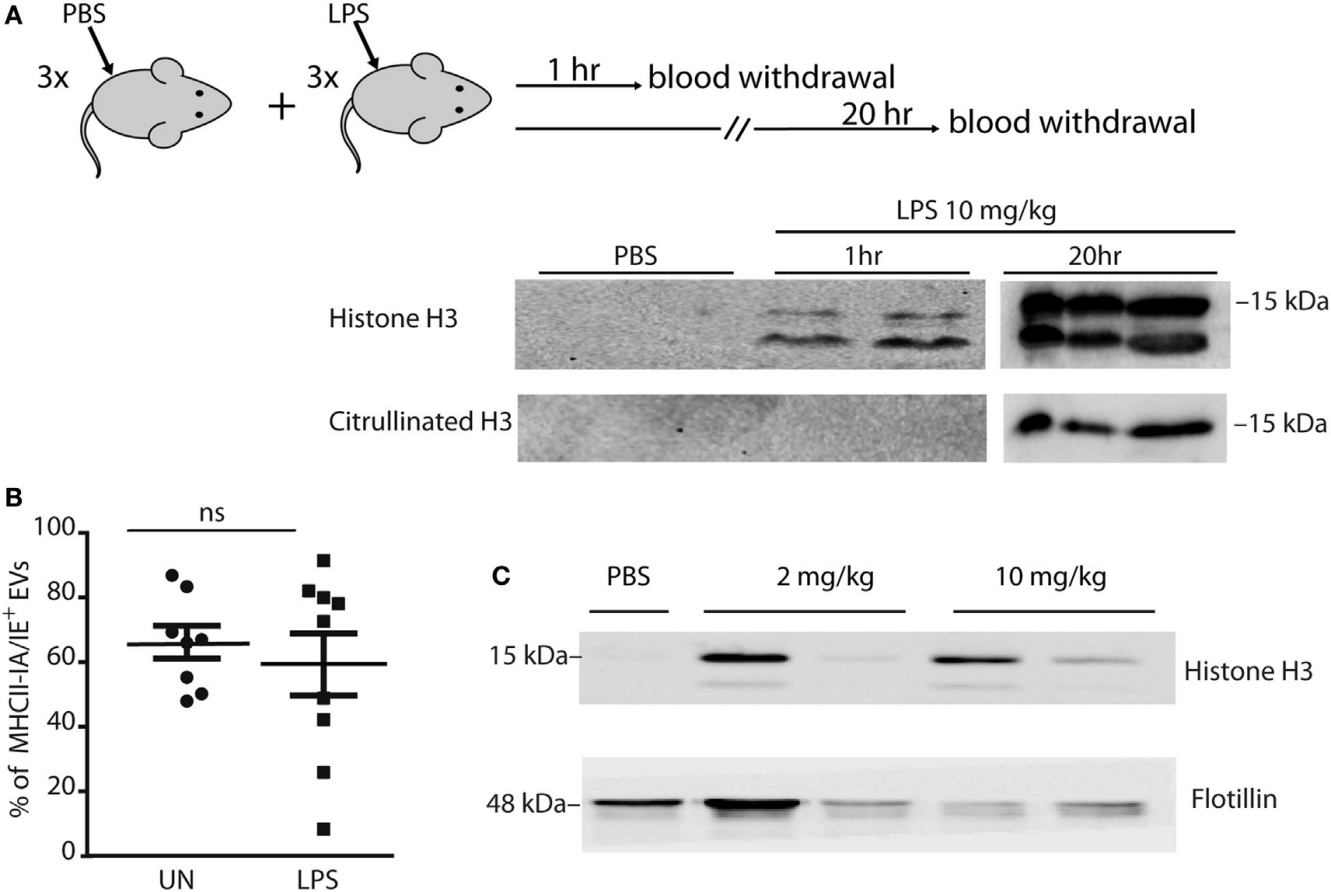
Mice challenged with LPS have histone-laden extracellular vesicles (EVs) in plasma. **(A)** Mice (*n* = 3 per group) were injected in the tail vein either with PBS or 10 mg/kg LPS, and blood was withdrawn 1 and 20 h after injection. Western blot analysis of histone H3 and citrullinated histone H3 in plasma. Each lane contains plasma (20 µl) from one mouse. **(B)** Mice (*n* = 9 per group) were injected in the tail vein either with PBS or 10 mg/kg LPS, and blood was withdrawn 1 h later. EVs in plasma were trapped on anti-CD63-coated beads, stained with PE-labeled antibody against the MHCII IA/IE marker, and analyzed by flow cytometry. The graph shows the fraction of IA/IE positive beads in plasma from individual mice. Averages and SDs are indicated. **(C)** Mice were injected in the tail vein either with PBS, 2 or 10 mg/kg LPS, and blood was withdrawn 1 h later. All mice injected with 10 mg/kg LPS died within the same day, all other mice survived. CD63-positive EVs were trapped with beads, heated in SDS-PAGE denaturation buffer, and histone H3 was assessed by Western blotting. This experiment was performed twice with similar results (see Figure S4 in Supplementary Material).

Mice challenged with lethal amounts of LPS (10 mg/kg) have circulating histones in blood (3). To determine if some of these histones are associated to vesicles deriving from monocytes/macrophages, we injected mice with 10 mg/kg LPS and assessed the levels of histones in plasma 1 and 20 h later. We found significant levels of circulating H3 histone already 1 h postinjection, which increased at 20 h (Figure 5A). Remarkably, citrullinated H3 histone (deriving from neutrophils) appeared only at the later time, indicating that NETs from neutrophils contribute to circulating histones, as expected, but with a delayed kinetics consistent with the timing of neutrophil activation and NET production (23).

To isolate EVs, we incubated plasma from unchallenged and LPS-challenged mice with anti-CD63 coated beads, which were then stained with PE-labeled antibodies against IA/IE (a MHC Class II antigen that is present on antigen-presenting cells including monocytes/macrophages). We verified that the number of positive beads was proportional to the amount of EVs by diluting the plasma before adding it to same amount of beads (Figure S3 in Supplementary Material).

Flow cytometry indicated that between 80 and 90% of the beads were positive for IA/IE, with no significant difference between LPS-injected and control (PBS-injected) mice (Figure 5B; Figure S4A in Supplementary Material).

We repeated the experiment by injecting mice with either lethal (10 mg/kg) or sublethal (2 mg/kg) amounts of LPS, and again found that the amount of vesicles (assessed *via* the flotillin marker) was variable but non-zero in all conditions, whereas only the EVs from LPS-injected mice were loaded with histones (Figure 5C).

### Histones Are Present Both on the Outer Surface and Inside the EVs

**Figure 6:**
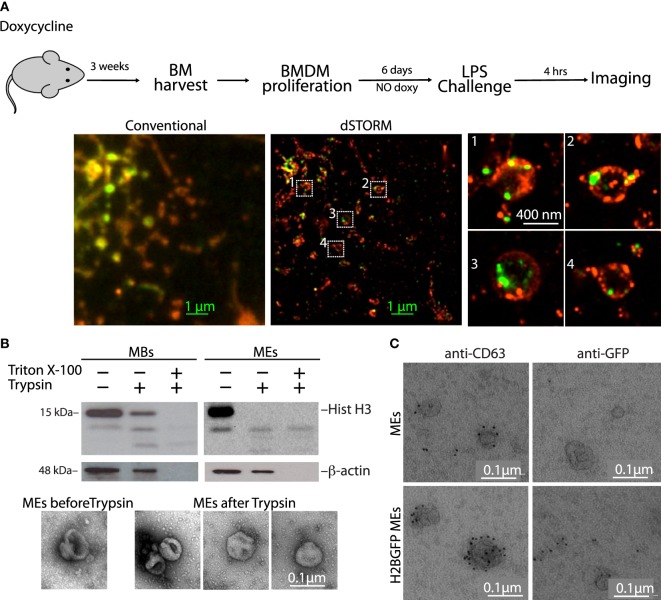
Histones are present on the outer surface of microvesicles and exosomes (MEs). **(A)** H2B-GFP mice were exposed to doxycycline to induce H2B-GFP expression. Three weeks later, the mice were sacrificed and their bone marrows were harvested and cultured without doxycycline. In this way, bone marrow-derived macrophages (BMDMs) only contained H2B-GFP synthesized several weeks earlier, and all H2B-GFP was chromatin-associated. H2B-GFP BMDMs were then challenged with LPS, and imaged by stochastic optical reconstruction microscopy. H2B-GFP is green; cell membranes were stained red with concanavalin A-TRITC. Zooms of the regions inside the white squares are shown. **(B)** Western blot of macrobodies and MEs treated with trypsin in the presence or not of detergent (Triton X-100). The electron micrographs show representative MEs before and after trypsinization. **(C)** Transmission electron microscopy images of exosomes from LPS-challenged BMDMs, either from wt or H2B-GFP mice, immunostained for CD63 and GFP. Scale bars: 0.1 µm. Figure S6 in Supplementary Material shows electron micrographs of extracellular vesicles immunostained for histone H2A and H3.

We next visualized vesicles from LPS-activated BMDMs with super resolution microscopy. We used H2B-GFP macrophages, staining membranes with concanavalin A-TRITC (Figure 6A). H2B-GFP colocalized with a large fraction of vesicles, ranging from 100 to 400 nm.

To understand whether histones were associated with the outer leaflet of the vesicle membrane or were protected inside the vesicle, we used a biochemical approach. We incubated the vesicles with trypsin, in the presence or absence of Triton X-100. In MBs, all H3 histone was accessible to trypsin in the presence of detergent, whereas in the absence of detergent, a fraction was not digested, indicating that histones were present both within and on the surface of the vesicles (Figure 6B). In the ME fraction, H3 histone was completely degraded even in the absence of detergent, indicating that it was only present on the outer surface of the vesicles. Beta-actin, which also was found within both MBs and EVs, is accessible only in the presence of detergent. Electron microscopy analyses confirmed that trypsin digestion did not destroy MEs (Figure 6B, bottom).

To further confirm these results, we visualized by electron microscopy EVs immunostained for GFP and CD63. We found H2B-GFP and CD63 on the outer surface of vesicles from LPS-activated H2B-GFP BMDMs, whereas no GFP staining was detected on the surface of vesicles from wt BMDMs (Figure 6C).

### Soluble Histones and EVs Originating From LPS-Exposed BMDMs Induce Inflammatory Responses

Numerous reports indicate that histones cause inflammatory responses (3–6). We then asked whether the different histone fractions (soluble, MBs, and MEs) originating from LPS-exposed BMDMs could elicit inflammatory responses in naïve BMDMs, using transcription of cytokine genes (Tnf, IL-1β, and IL-6) as a readout.

As a preliminary experiment, we showed that LPS elicited a strong transcriptional response in our assay, and that PMB could suppress about 99% of this response (Figure S5 in Supplementary Material). We then added PMB in all subsequent experiments to ensure that the inflammatory response was not due to LPS carried over in the samples.

**Figure 7:**
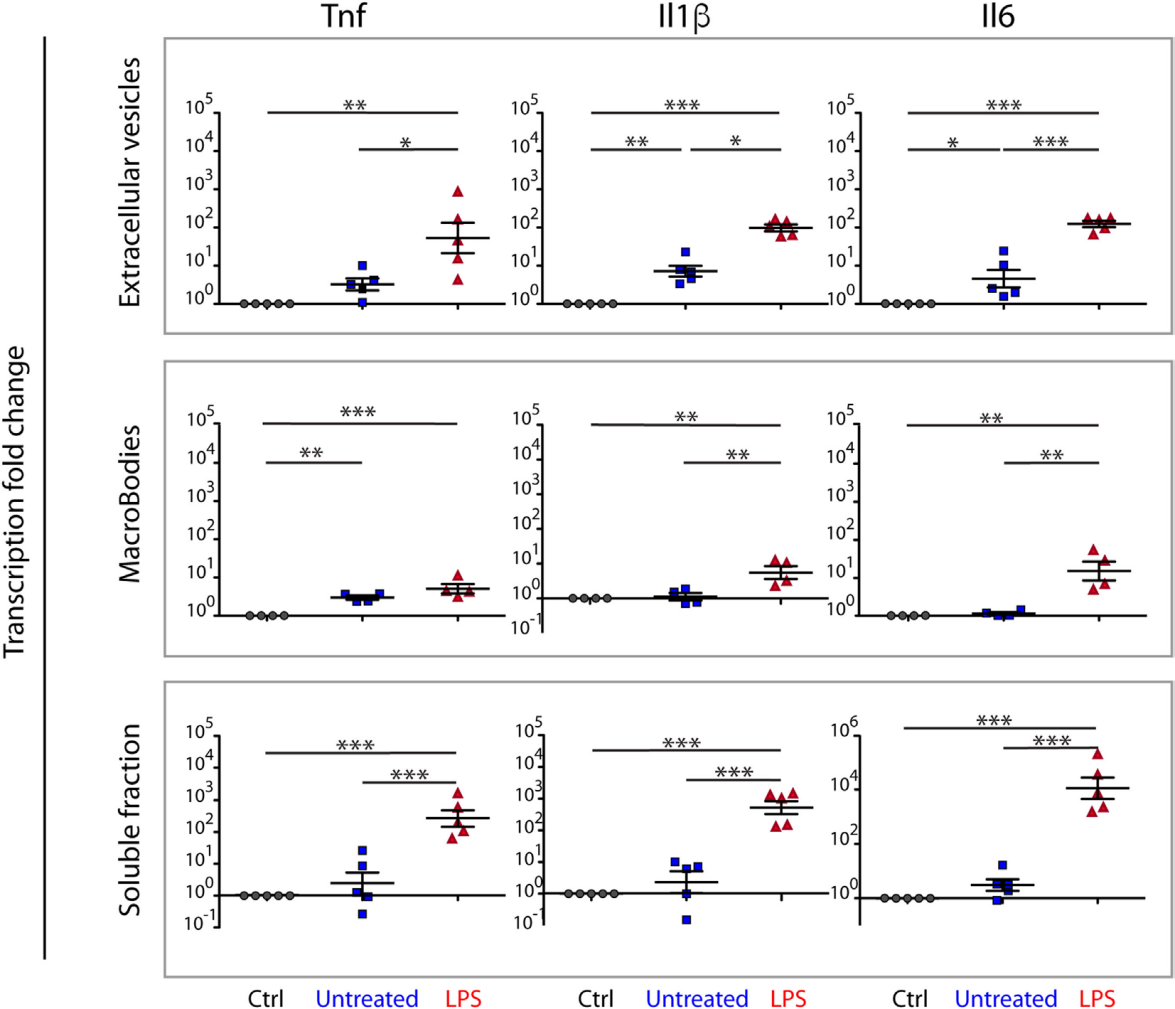
Soluble, MB, and ME-associated molecules from LPS-challenged bone marrow-derived macrophages (BMDMs) induce inflammatory responses on naive BMDMs. Naïve BMDMs were incubated for 4 h with aliquots of the fractions obtained after density centrifugation of the CCM from unchallenged BMDMs (untreated), LPS-challenged BMDMs (LPS), or BMDMs incubated with an equivalent volume of PBS (Crtl). Fractions containing soluble proteins, macrobodies, or microvesicles and exosomes and were tested separately. The inflammatory response was assayed as transcription of inflammatory genes (*Il6, Il1b*, and *Tnf*), measured by real-time qPCR. Each dot represents a biological replicate. Averages and SDs are indicated. Statistical significance was tested with one-way ANOVA with Tukey’s posttest; *p < 0.05, **p < 0.01, ***p < 0.001.

Both the soluble and the ME fractions from LPS-challenged BMDMs elicited strong inflammatory responses when compared to the same fractions from untreated BMDMs. The MB fraction elicited comparatively weak inflammatory responses when compared to those from the ME and soluble fractions (Figure 7).

**Figure 8:**
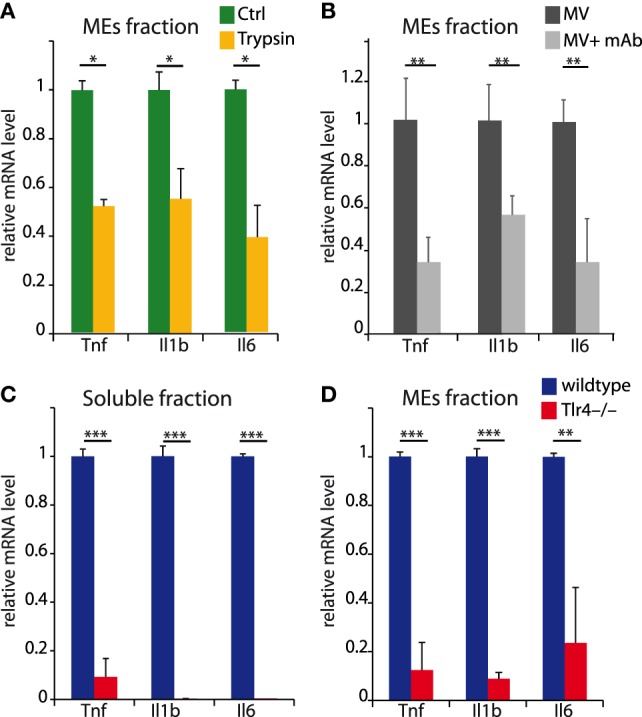
Soluble and microvesicles and exosomes (MEs)-associated histones induce inflammation *via* toll-like receptor 4 (TLR4). **(A,B)** MEs were incubated or not with trypsin **(A)** or a monoclonal antibody binding all histones **(B)**, and used to induce inflammatory responses on naïve bone marrow-derived macrophages (BMDMs) (see Figure 7). **(C,D)** BMDMs (either from wt or *Tlr4^−/−^* mice) were incubated for 4 h with aliquots of the soluble **(C)** or MEs fraction **(D)** obtained after density centrifugation of the supernatants of BMDMs challenged with LPS. In both panels, mRNA levels of inflammatory genes were measured by real-time qPCR and compared using *t* tests; *p < 0.05, **p < 0.01, ***p < 0.001. Error bars represent SDs.

This experiment, however, does not directly assign the inflammatory activity of MEs from LPS-challenged BMDMs to histones. Thus, we incubated the ME fraction with trypsin before adding it to naïve BMDMs and showed that trypsin strongly reduced the inflammatory activity (Figure 8A). Likewise, a monoclonal antibody reported to inhibit all histones (mAb clone H11-4) strongly reduced the inflammatory activity of the ME fraction (Figure 8B).

Histones have been shown to activate macrophages *via* TLR4. We, therefore, exposed BMDMs obtained from *Tlr4^−/−^* mice to the fractions containing soluble histones, MB, or MEs, and compared their transcriptional responses to those of BMDMs from wild-type mice. As expected, the responses of *Tlr4^−/−^* BMDMs to both fractions was limited or absent (Figures 8C,D).

Taken together, these experiments show that vesicle-associated histones can interact with receptors on bystander cells, notably with TLR4.

## Discussion

Circulating histones have been identified as mediators of damage in animal models of sepsis (3) and in patients with sepsis (5, 24). Circulating histones are presumed to derive primarily from neutrophils and the NETs they produce, and damaged or apoptotic cells from the endothelia or other tissues. Here, we show that a part of the circulating histones in septic mice derives from monocytes/macrophages, which release them on EVs without dying. We do confirm that some circulating histones come from neutrophil NETs, as they are citrullinated; however, these appear rather late after LPS inoculation of mice. Remarkably, histones associated with the fraction containing MEs are found on their external surface and can activate inflammatory receptors on target cells, and in particular, macrophages/monocytes.

We started from the previously published observation that macrophages activated with LPS lose 20% of their nuclear histones (7). Here, we investigated whether the “missing” histones were degraded or released from the activated macrophages. We showed by super-resolution microscopy that chromatin is globally reorganized in the nucleus of LPS-challenged BMDMs following histone loss. We could not find any evidence of death of LPS-challenged BMDMs, either by annexinV-PI staining, TUNEL assay, or LDH release. Indeed, LPS appeared to limit BMDM cell death. LPS has protective antiapoptotic effects on cardiomyoctes, monocytes/macrophages, and neutrophils (25–27), and induces MyD88-dependent survival in macrophages (22). Possibly, the global unpacking of chromatin favors the expression of the vast number or genes whose transcription is activated upon LPS stimulation of BMDMs, including those for survival.

Contrary to our expectations, we found that LPS-activated macrophages do not degrade histones, but rather release them into the extracellular medium. Histones can be degraded by the proteasome (18), but the proteasome inhibitor MG132 did not prevent the histone decrease in activated BMDMs. Conversely, we found histones in the extracellular medium, both in soluble and vesicle-associated form. We demonstrated that histones are associated to vesicles by differential centrifugation, electron microscopy and super-resolution optical microscopy, and vesicle capture with beads coated with antibodies directed against CD63, an integral membrane protein. We recovered vesicle-associated histones both from wt BMDMs (as shown by western blotting) and from H2B-GFP BDMSs (as shown by flow cytometry). In fact, we recovered vesicle-associated histones also from the plasma after the mice received lethal doses of LPS. Remarkably, histones were found in most proteomic analyses of EVs from various cell types [(28); http://www.exocarta.org/] but, to our knowledge, the specificity and significance of their presence was never investigated before. We do not know whether soluble histones, which are also released by activated macrophages, are secreted *via* a non-classical secretion route, or simply detach from the surface of EVs.

The vesicle-associated histones bear classical posttranslational modifications associated with both transcriptionally active and inactive chromatin, such as H3K36 trimethylation and H3K9 trimethylation, respectively. This suggests that these histones come from the nuclear pool. However, we cannot exclude that some of the histones that are synthetized in the first hour following LPS-stimulation (when the histone content in BMDMs increases, Figure 1) are directly translocated to vesicles directed for secretion rather than being deposited onto chromatin.

The most notable feature of ME-associated histones is their topological localization, on the outer membrane of the vesicle. This allows for the exposure of histones to surface receptors of target cells, and indeed, we have shown that histone-bearing MEs can activate inflammatory responses in other BMDMs, and potentially on all cells bearing TLR4. Notably, MEs, which expose histones on the outer surface, are more inflammatory than MBs, where only part of the histones are external. Indeed, MEs can present a locally elevated concentration of histones, acting as DAMPs, to nearby cells. In this way, histone-bearing MEs could amplify and spread the inflammatory signals emitted by BMDMs encountering LPS (or rather, Gram-negative bacteria). We speculate that histone-bearing EVs might even contribute to the formation of anti-histone antibodies in autoimmune diseases.

We have shown that macrophage-derived EVs with associated histones are abundant in the plasma after the mice receive high doses of LPS, which indicates that such EVs can attain systemic dissemination. We cannot precisely evaluate the proportion of extracellular histones deriving from monocytes/macrophages in comparison to other sources, for example, dead cells or NETs produced by neutrophils, and in fact, this proportion might vary extensively depending on the prevailing local and systemic conditions. However, most macrophages decrease their content of nuclear histones upon activation, and we infer that most activated macrophages will release in their environment EV-associated histones. Thus, the emission of EV-associated histones appears to be yet another specific activity associated to macrophage activation and is most likely endowed with positive adaptive value, like the extrusion of chromatin in NETs by activated neutrophils.

In our work, we focused on macrophages. However, most cell types produce EVs, and all nucleated cell types contain histones. Future work will determine whether other cell types modulate their histone content following diverse stimuli and generate EVs with associated histones, at least in some conditions and in specific microenvironments.

The extracellular release of histones is highly unexpected, but we note that this process can at the same time increase the local accessibility of DNA in the genome and allow cells to communicate a state of infection or danger to bystander cells, perhaps even systemically.

## Ethics Statement

Mice were housed in the SPF animal facility at San Raffaele Scientific Institute with access to food and water *ad libitum*. All experimental protocols were approved by the San Raffaele Institutional Animal Care and Use Committee (IACUC 838) in accordance with the Italian Ministry of Health. All efforts were made to minimize suffering.

## Author Contributions

RN, MB, and AA conceived and designed the experiments; RN, FB, AG, and DM performed the experiments; RN, FB, DM, AA, and MB analyzed the data; AA and MB wrote the paper.

## Conflict of Interest Statement

The authors declare that the research was conducted in the absence of any commercial or financial relationships that could be construed as a potential conflict of interest.

## Supplementary Material

Figure S1 MG132 abrogates LPS-induced transcription of *Ikbia, Il6, Il1b*, and *Tnf* genes. Bone marrow-derived macrophages (BMDMs) from wt mice were left unstimulated or incubated for 4 h with LPS, MG132, or the combination thereof. qPCR performed on *Ikbia, Il6, Il1b*, and *Tnf* genes showed that transcription was decreased by two to three orders of magnitude in BMDMs incubated with MG132 + LPS as compared to LPS alone. *X*-axis, fold change of transcription in logarithmic scale. The table at the bottom shows the actual fold changes. Transcription of the untreated sample is set to 1.

Figure S2 Flow cytometry and electron microscopy analysis of macrobodies (MBs) and microvesicles and exosomes (MEs). **(A)** FACS analysis of MBs. Reference beads with defined size (1, 3, and 6 µm: black, blue, and red, respectively) were run together with MBs to define the size scale. MEs are too small and cannot be distinguished from the electronic noise of the FACS instrument (data not shown). **(B)** Representative electron microscopic images of MBs and MEs. Bars, 0.5 µm.

Figure S3 Validation of the assay to measure the number of IA/IE-positive extracellular vesicles. Plasma from an untreated mouse was diluted with PBS and incubated with anti-CD63 coated beads; beads were then stained with PE-labeled antibody against IA/IE (a marker of antigen-presenting cells). The number of IA/IE positive beads is proportional to the amount of vesicles in the diluted plasma. This assay was the used to quantify vesicles in the plasma of mice challenged or not with LPS (Figure 5).

Figure S4 Histone-laden extracellular vesicles (EVs) in plasma from mice challenged with LPS. **(A)** Flow cytometry data on the number of IA/IE positive EVs in plasma of PBS- or LPS-injected mice. Plasma was diluted with PBS and incubated with anti-CD63 coated beads as described in the legend to Figure S3 in Supplementary Material. Shown are representative data from six mice out of 18; the abridged data from all 18 mice are shown in Figure 5C. **(B)** Replica of the experiment shown in Figure 5B. Mice (*n* = 3 per group) were injected in the tail vein either with PBS or 10 mg/kg LPS, and blood was withdrawn 1 and 20 h after injection. Western blot analysis of histone H3 and citrullinated histone H3 in plasma. Each lane contains plasma (20 µl) from one mouse. **(C)** Replica of the experiment shown in Figure 5C. Mice were injected in the tail vein either with PBS or 10 mg/kg LPS, and blood was withdrawn 1 h later. All mice injected with 10 mg/kg LPS died within the same day, all other mice survived. CD63-positive EVs were trapped with beads, heated in SDS-PAGE denaturation buffer, and histone H3 was assessed by Western blotting.

Figure S5 Polymyxin B (PMB) abrogates the activity of LPS present in the medium of LPS-challenged BMDMs. qPCR shows that transcriptional activity induced by LPS is reduced 200–1,000-fold by PMB. This allows to add LPS to BMDMs, but then eliminate its activity when the supernatant of LPS-challenged BMDMs is tested on naïve BMDMs (Figure 7). Groups are compared using one-way ANOVA plus posttests; ****p* < 0.001. Averages and SDs are indicated.

Figure S6 Electron microscopy imaging reveals positivity for histones in microvesicles and exosomes (MEs) and macrobodies (MBs). A mixed population of MBs and MEs was stained with immunogold conjugated antibodies anti-H2A (upper panels) or anti-H3 (lower panels) and analyzed by EM. Scale bar: 0.5 µm.
