# Supplementary material for: LPS-Challenged Macrophages Release Microvesicles Coated With Histones
Source: Front Immunol. 2018 Jun 27;9:1463. doi: 10.3389/fimmu.2018.01463 (PMC6030250; doi:10.3389/fimmu.2018.01463)
Supplement: Figure S1 — MG132 abrogates LPS-induced transcription of Ikbia, Il6, Il1b, and Tnf genes. Bone marrow-derived macrophages (BMDMs) from wt mice were left unstimulated or incubated for 4 h with LPS, MG132, or the combination thereof. qPCR performed on Ikbia, Il6, Il1b, and Tnf genes showed that transcription was decreased by two to three orders of magnitude in BMDMs incubated with MG132 + LPS as compared to LPS alone. X-axis, fold change of transcription in logarithmic scale. The table at the bottom shows the actual fold changes. Transcription of the untreated sample is set to 1. [file image_1.tif]

Nair et al., 2018

Supplementary Figure 1:

MG132 abrogates LPS induced transcription of Tnf, Il1b, Il6 and Nfkbia genes

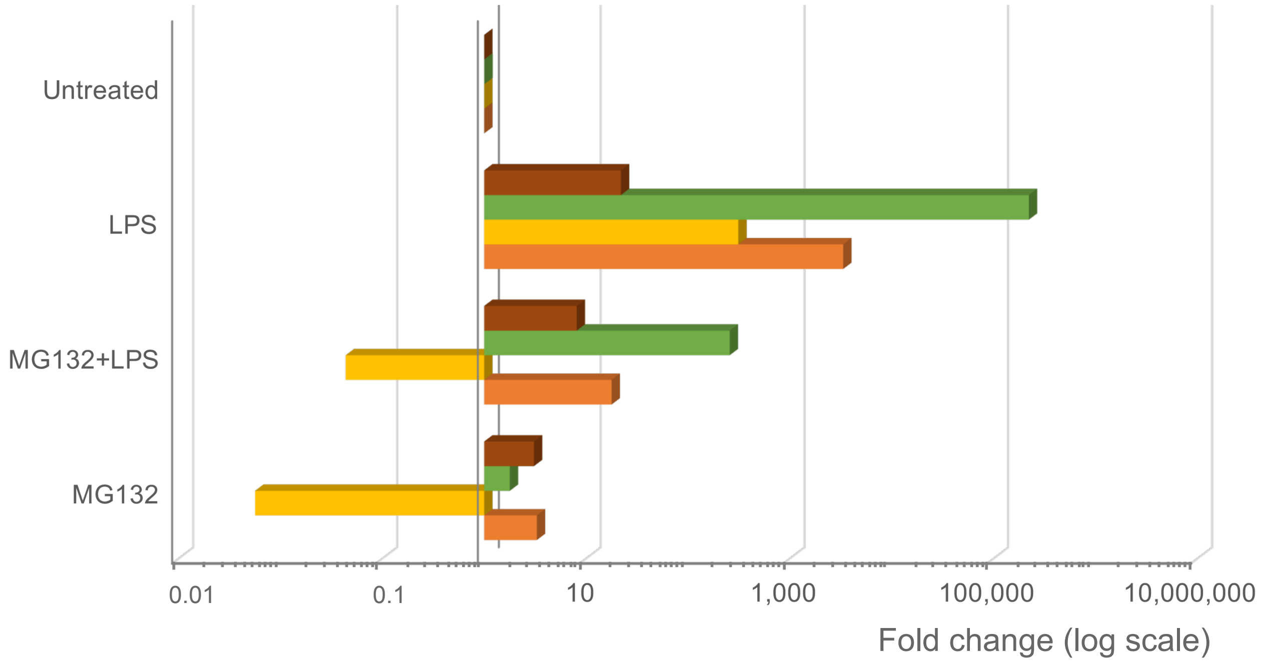

	MG132	MG132+LPS	LPS	Untreated
Nfkbia	3.1	8.1	22.0	1.0
IL6	1.8	257.2	221973.3	1.0
Il1b	0.0	0.0	312.6	1.0
Tnf	3.3	17.8	3344.4	1.0
