# Supplementary figures and images for: LPS-Challenged Macrophages Release Microvesicles Coated With Histones
Source: Front Immunol. 2018 Jun 27;9:1463. doi: 10.3389/fimmu.2018.01463 (PMC6030250; doi:10.3389/fimmu.2018.01463)

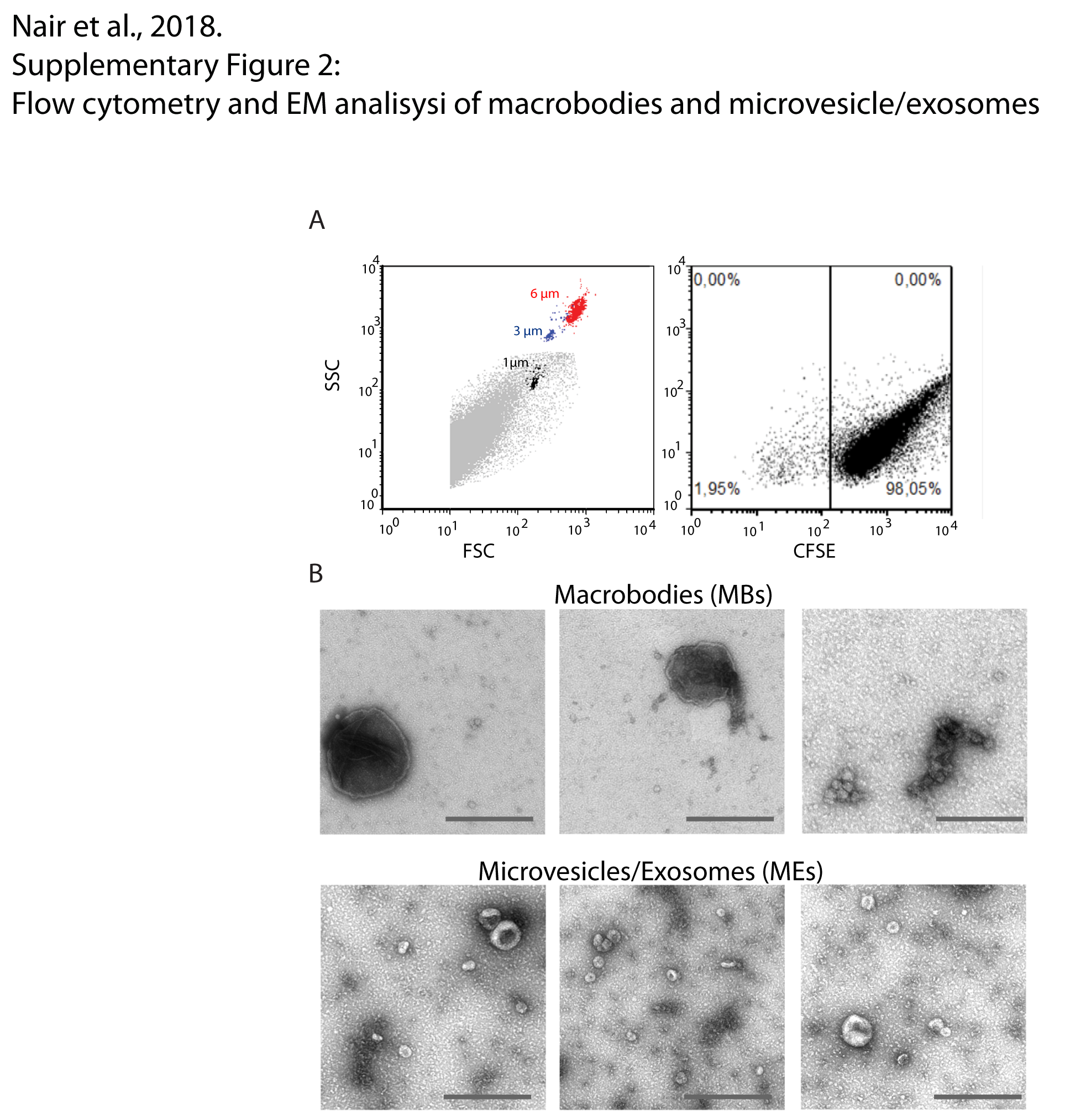

Supplement: Figure S2 — Flow cytometry and electron microscopy analysis of macrobodies (MBs) and microvesicles and exosomes (MEs). (A) FACS analysis of MBs. Reference beads with defined size (1, 3, and 6 µm: black, blue, and red, respectively) were run together with MBs to define the size scale. MEs are too small and cannot be distinguished from the electronic noise of the FACS instrument (data not shown). (B) Representative electron microscopic images of MBs and MEs. Bars, 0.5 µm. [file image_2.tif]

Supplementary Figure 4:  
Mice challenged with LPS have histone-laden EVs in plasma.

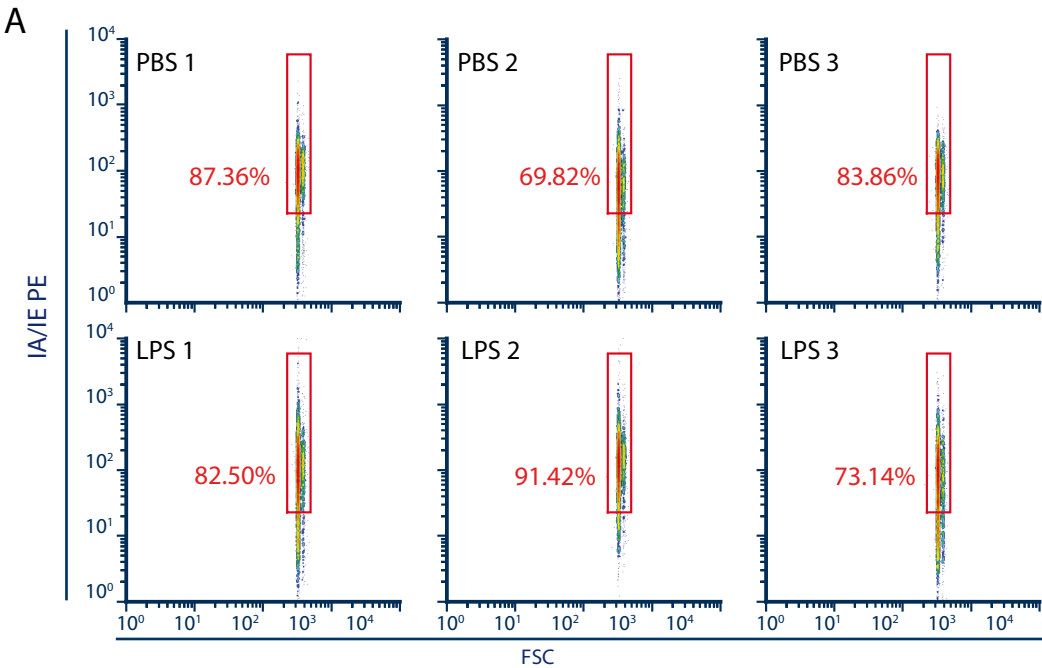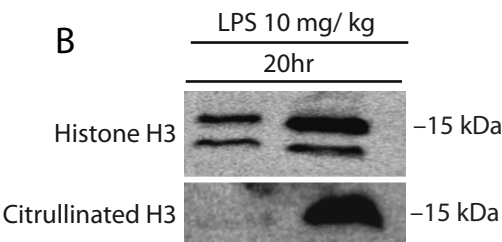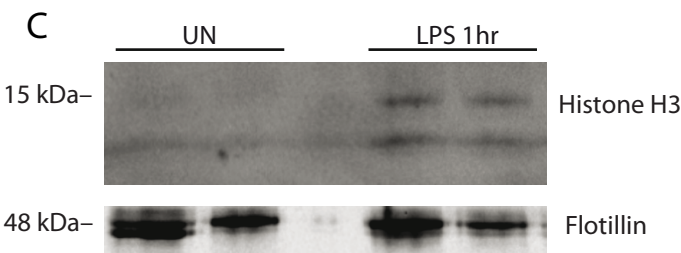

Supplement: Figure S4 — Histone-laden extracellular vesicles (EVs) in plasma from mice challenged with LPS. (A) Flow cytometry data on the number of IA/IE positive EVs in plasma of PBS- or LPS-injected mice. Plasma was diluted with PBS and incubated with anti-CD63 coated beads as described in the legend to Figure S3 in Supplementary Material. Shown are representative data from six mice out of 18; the abridged data from all 18 mice are shown in Figure 5C. (B) Replica of the experiment shown in Figure 5B. Mice (n = 3 per group) were injected in the tail vein either with PBS or 10 mg/kg LPS, and blood was withdrawn 1 and 20 h after injection. Western blot analysis of histone H3 and citrullinated histone H3 in plasma. Each lane contains plasma (20 µl) from one mouse. (C) Replica of the experiment shown in Figure 5C. Mice were injected in the tail vein either with PBS or 10 mg/kg LPS, and blood was withdrawn 1 h later. All mice injected with 10 mg/kg LPS died within the same day, all other mice survived. CD63-positive EVs were trapped with beads, heated in SDS-PAGE denaturation buffer, and histone H3 was assessed by Western blotting. [file image_4.tif]
