# Supplementary material for: LPS-Challenged Macrophages Release Microvesicles Coated With Histones
Source: Front Immunol. 2018 Jun 27;9:1463. doi: 10.3389/fimmu.2018.01463 (PMC6030250; doi:10.3389/fimmu.2018.01463)

Nair et al., 2018

Supplementary Figure 3:

Validation of the assay to measure the number of IA/iE positive Evs

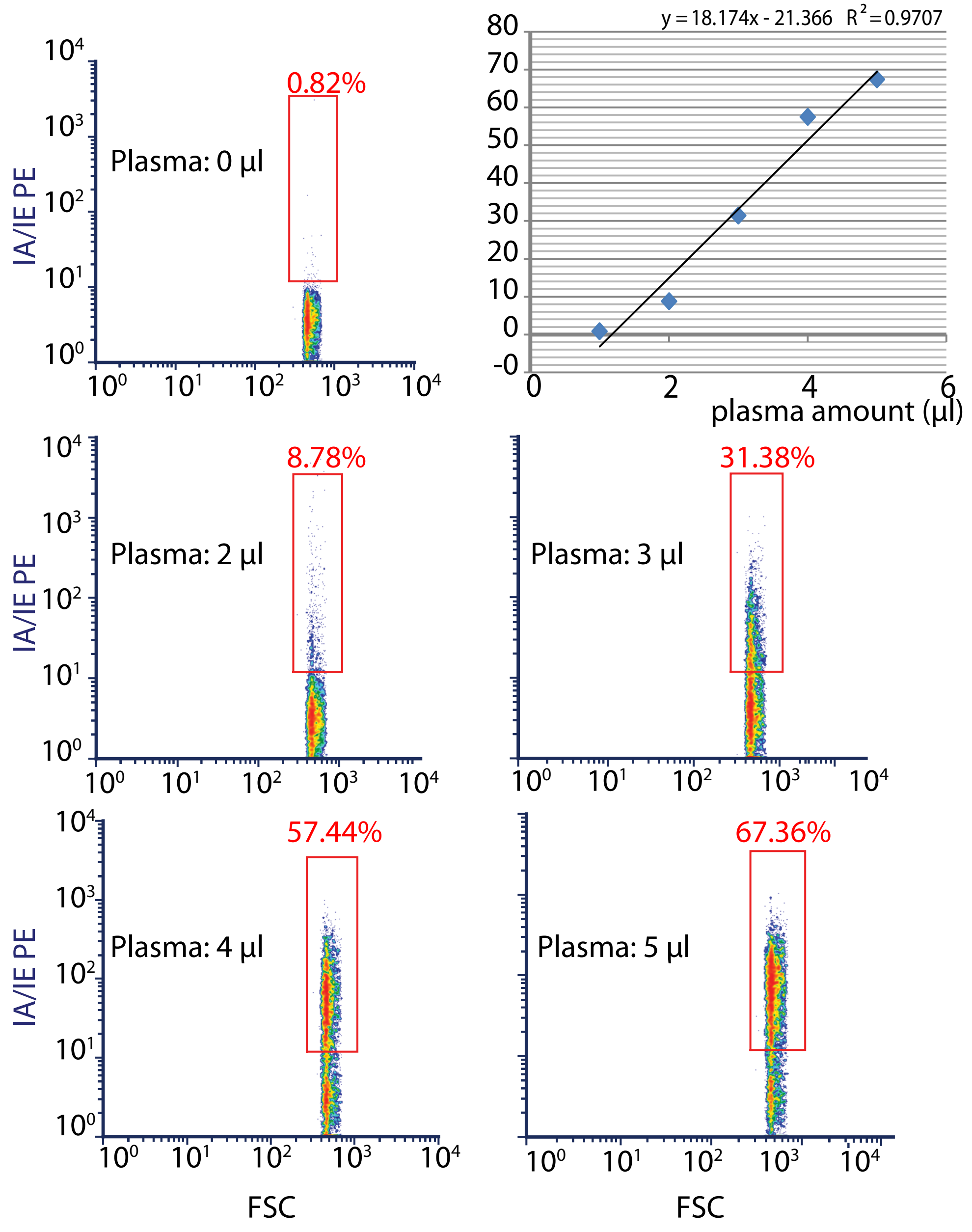

Supplement: Figure S3 — Validation of the assay to measure the number of IA/IE-positive extracellular vesicles. Plasma from an untreated mouse was diluted with PBS and incubated with anti-CD63 coated beads; beads were then stained with PE-labeled antibody against IA/IE (a marker of antigen-presenting cells). The number of IA/IE positive beads is proportional to the amount of vesicles in the diluted plasma. This assay was the used to quantify vesicles in the plasma of mice challenged or not with LPS (Figure 5). [file image_3.tif]
