# Supplementary material for: LPS-Challenged Macrophages Release Microvesicles Coated With Histones
Source: Front Immunol. 2018 Jun 27;9:1463. doi: 10.3389/fimmu.2018.01463 (PMC6030250; doi:10.3389/fimmu.2018.01463)

Nair et al., 2018
Supplementary Figure 5:
PolymyxinB (PMB) abrogates the transcriptional activity of LPS on inflammatory genes by 200-1000 fold in the transcription assay used in Figure 7.

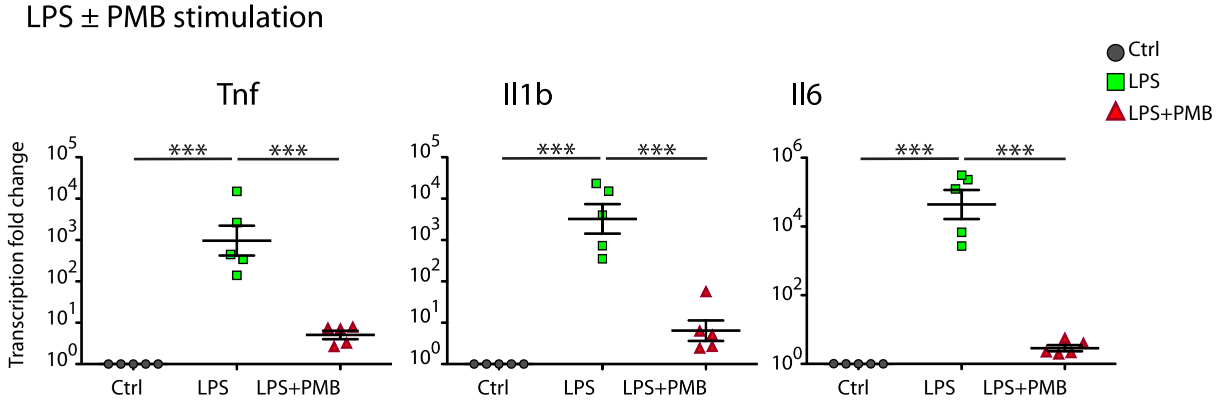

Supplement: Figure S5 — Polymyxin B (PMB) abrogates the activity of LPS present in the medium of LPS-challenged BMDMs. qPCR shows that transcriptional activity induced by LPS is reduced 200–1,000-fold by PMB. This allows to add LPS to BMDMs, but then eliminate its activity when the supernatant of LPS-challenged BMDMs is tested on naïve BMDMs (Figure 7). Groups are compared using one-way ANOVA plus posttests; ***p < 0.001. Averages and SDs are indicated. [file image_5.tif]
