# Supplementary material for: LPS-Challenged Macrophages Release Microvesicles Coated With Histones
Source: Front Immunol. 2018 Jun 27;9:1463. doi: 10.3389/fimmu.2018.01463 (PMC6030250; doi:10.3389/fimmu.2018.01463)

Nair et al, 2108

Supplementary Figure5: EM images of MEs mixed to MBs stained with immuno-gold conjugated antibodies against histones H2A and H3. Scale bar : 0.5 μm.

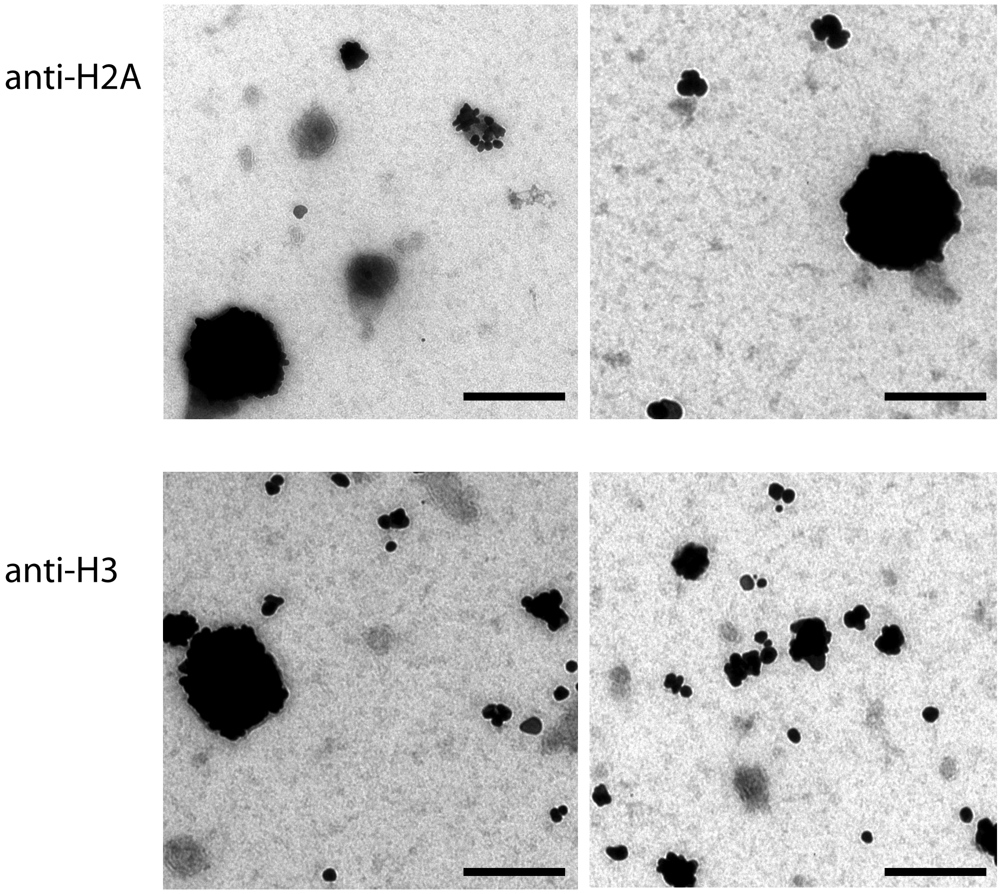

Supplement: Figure S6 — Electron microscopy imaging reveals positivity for histones in microvesicles and exosomes (MEs) and macrobodies (MBs). A mixed population of MBs and MEs was stained with immunogold conjugated antibodies anti-H2A (upper panels) or anti-H3 (lower panels) and analyzed by EM. Scale bar: 0.5 µm. [file image_6.tif]
